# Uncovering the Role of Surface-Attached Ag Nanoparticles in Photodegradation Improvement of Rhodamine B by ZnO-Ag Nanorods

**DOI:** 10.3390/nano12162882

**Published:** 2022-08-22

**Authors:** Svetlana Em, Mussa Yedigenov, Laura Khamkhash, Shanazar Atabaev, Anara Molkenova, Stavros G. Poulopoulos, Timur Sh. Atabaev

**Affiliations:** 1 Department of Chemistry, School of Sciences and Humanities, Nazarbayev University, Nur-Sultan 010000, Kazakhstan; 2 Department of Professional Disciplines, Academy of the Ministry of Emergency Situations, Tashkent 100102, Uzbekistan; 3 Institute of Advanced Organic Materials, Pusan National University, Busan 46241, Korea; 4 Department of Chemical and Materials Engineering, School of Engineering and Digital Sciences, Nazarbayev University, Nur-Sultan 010000, Kazakhstan

**Keywords:** ZnO nanorods, Ag nanoparticles, photodegradation, photocatalytic activity, radical scavengers, rhodamine B

## Abstract

ZnO nanorods decorated with metal nanoparticles have sparked considerable interest in recent years thanks to their suitability for a wide range of applications, such as photocatalysis, photovoltaics, antibacterial activity, and sensing devices. In this study, we prepared and investigated the improved solar-light-assisted photocatalytic activity of ZnO nanorods (NRs) decorated with Ag nanoparticles (NPs) using a conventional rhodamine B (RB) dye as a model water pollutant. We showed that the presence of Ag NPs on the surface of ZnO NRs significantly increases the degradation rate of RB dye (~0.2432 min^−1^) when compared to bare ZnO NRs (~0.0431 min^−1^). The improved photocatalytic activity of ZnO-Ag was further experimentally tested using radical scavengers. The obtained results reveal that **˙**OH and **˙**O_2_^−^ radicals are main active species involved in the RB dye photodegradation by ZnO-Ag NRs. It was concluded that efficient charge separation plays a major role in photocatalytic activity improvement.

## 1. Introduction

Toxic organic pollutants released from the textile industry into water bodies cause serious environmental problems, such as water, soil, and aquatic life poisoning. For example, organic azo dyes are particularly hazardous due to their toxicity, relative stability, and carcinogenicity [1]. Light-assisted photocatalytic dye degradation using metal oxide nanostructures is currently considered as an effective and promising technique for colored wastewater treatment. Typically, highly reactive hydroxyl radicals (**˙**OH) are formed during this process, which can oxidize virtually any organic compound present in water [2,3]. 

Various metal oxides have been routinely tested as photocatalysts in dye degradation experiments to date. Among them, ZnO semiconductor has attracted the scientific community’s interest due to its low price, ability to control the size and shape, and the possibility to use it in a broad range of applications, including photocatalysis [4,5], dye and pesticides removal [6,7], gas sensing [8], water splitting [9], and so on. On the other hand, ZnO has a high bandgap (E_g_~3.37 eV) and can thus be activated by UV photons only. In this regard, ZnO is often sensitized with plasmonic metallic NPs such as Ag, Au, and Pt to improve the absorption of the photons in the visible light region. Owing to their lower cost, Ag NPs are more commonly used as compared to Au or Pt NPs. There are a number of studies available in the literature that describe the preparation and use of ZnO-Ag nanostructures. ZnO-Ag nanostructures, for example, have been found useful for water disinfection [10], as antibacterial agents [11], and for photodegradation of organic pollutants in water, such as 4-nitrophenol [12,13] and organic dyes [14,15,16]. It is widely accepted that charge separation plays an important role in improving the photocatalytic activity of ZnO-Ag nanostructures; however, this statement is typically tested solely through the photoluminescence (PL) study [14,16], which is not enough for a complete description of the photocatalytic mechanism. For example, PL analysis showed a similar trend for two recent works [15,17], i.e., PL peaks of intensity decreased, implying better charge separation in ZnO-Ag structures. Given the very similar preparation methodology of ZnO-Ag, one would expect photocatalytic mechanisms to be similar; however, it was not confirmed in practice. For instance, X. Zhu and colleagues [15] revealed that **˙**O_2_^−^ radicals are mainly responsible for the photodegradation process, whereas A.N. Kadam and colleagues [17] showed that the main reactive species involved in dye degradation were **˙**OH and **˙**O_2_^−^ radicals. Hence, the use of radical scavengers can be an effective method for elucidating the photodegradation mechanisms, but this methodology is rarely employed for metal oxide nanostructures sensitized with plasmonic nanostructures.

To the best of our knowledge, the photodegradation mechanism of ZnO NRs with chemically attached plasmonic nanostructures (without thermal treatment) has not yet been reported in the literature. Thus, the primary goal of this study is to fabricate ZnO NRs with chemically attached Ag NPs and uncover the role of Ag NPs in photocatalytic activity improvement. Another advantage of the proposed work is the use of a chemical modification method, which eliminates the need for high-temperature thermal treatment and reduces energy consumption/photocatalyst cost.

## 2. Materials and Methods

Zinc nitrate hexahydrate (98%), zinc acetate dihydrate (98.0%), hexamethylenetetramine (99.0%), silver nitrate (99.0%), rhodamine B dye (RB, ≥95.0%), (3-Aminopropyl) triethoxysilane (APTES, 99.0%), potassium iodide (99.0%), isopropanol (99.5%), p-benzoquinone (98.0%), and sodium borohydride (99.0%) were purchased from Merck KGaA (Darmstadt, Germany) and used as received.

Microscope glass slides were ultrasonically cleaned in ethanol (95–96%) for 5 min before drying naturally. The next step was to prepare a stock solution of zinc acetate dehydrate (5 mmol) in ethanol. Several droplets of stock solution were spin-coated for 15 s on clean glass slides at 2000 rpm. Later on, the glass slide was moved to a heated plate and maintained at 160 °C for 30 min to generate a thin ZnO seed layer. To ensure complete coverage of glass slides with a thin ZnO layer, the deposition and hot plate annealing procedures were repeated two times.

Hexamethylenetetramine (0.7 g) and zinc nitrate hexahydrate (1.49 g) were dissolved in 100 mL of deionized (DI) water and then heated in a convection oven (90 °C) for 1 h. The prepared glass slide was placed into the hot solution with the ZnO seed layer facing down, closed, and heated at 90 °C for an additional two hours. Finally, the glass slide was taken out, cleaned several times with DI water, and dried. With the help of a commercial blade, ZnO NRs were scratched away from the glass slide and collected for further use.

As-prepared ZnO NRs (15 mg) were ultrasonically dispersed in 5 mL DI water. Following that, 1 mL ethanol and 50 µL APTES were added to the mixture, which was agitated at 500 rpm for 20 min. As a result, APTES-modified ZnO NRs can be obtained [18]. Next, silver nitrate (2 mg) was added, and the mixture was left to mix for another 20 min. Finally, 50 µL of freshly prepared sodium borohydride aqueous solution (1 mg/mL) was added, and an immediate color change was observed. The mixture containing ZnO-Ag NRs was agitated for an additional 5 min before being centrifuged and dried overnight in a convection oven at 60 °C. 

Photocatalyst (2 mg, ZnO or ZnO-Ag) was dispersed in 6 mL of RB solution (1 × 10^−5^ M) and stirred for 30 min in the dark to achieve adsorption–desorption equilibrium. After reaching the equilibrium, the samples were illuminated for a predetermined period of time using an LCS-100 solar simulator (100 W, AM 1.5G filter, Newport-Spectra Physics GmbH, Darmstadt, Germany). The distance between the solar simulator and the bottom of the beaker was adjusted to 1 sun by a commercial silicon reference cell. Adsorption and dye concentration values were determined at λ_max_ = 554 nm using Genesys 50 UV-Vis spectrophotometer (Thermo Fisher Scientific Inc., Waltham, MA, USA). All experiments were repeated three times, and the mean values were used to draw the graphs. 

Scavenging solutions with a concentration of 2 mmol were prepared first, i.e., potassium iodide (KI) as hole capturer, isopropanol (IPA) as **˙**OH capturer, and p-benzoquinone (BQ) as **˙**O_2_^−^ capturer. Prior to illumination, 50 µL of scavenging species were added to a mixture of RB solution and photocatalyst. After illumination, the solutions were filtered, and RB dye concentration was determined using a UV-Vis spectrophotometer. 

A scanning electron microscope SEM (Auriga Crossbeam 540, Carl Zeiss, Oberkochen, Germany) equipped with energy-dispersive X-ray spectroscopy was used for morphological and elemental analyses (EDX, Aztec, Oxford Instruments, Abingdon, UK). The morphology of prepared samples was also observed using a transmission electron microscope (TEM, JEM2010F, JEOL Ltd., Tokyo, Japan). Rigaku SmartLab X-ray Diffractometer (XRD, Rigaku Corp., Tokyo, Japan) with a Cu Kα radiation source has been used to acquire X-ray diffraction patterns. The iCAP 6300 Duo inductively coupled plasma–optical emission spectrometry ICP-OES spectrometer (Thermo Fisher Scientific Inc., Waltham, MA, USA) was utilized to perform the elemental analysis of ZnO-Ag NRs.

## 3. Results and Discussion

**Figure 1:**
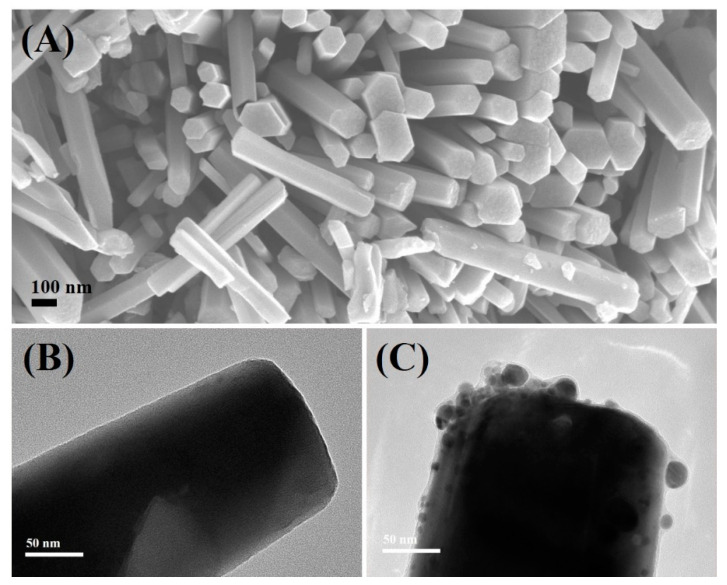
SEM (**A**) and TEM (**B**) images of as-prepared ZnO NRs. TEM image (**C**) of ZnO-Ag NRs (scale bar 50 nm).

APTES-modified ZnO NRs contain amino groups that can attract Ag ions/NPs to the surface, resulting in the formation of a composite structure as described elsewhere [19,20,21]. The use of a strong reducing agent such as sodium borohydride guarantees the formation of pure Ag NPs on ZnO surface. Figure 1A depicts an SEM image of as-prepared ZnO NRs. Size analysis revealed that the lengths of ZnO NRs were in the ~700–1100 nm range, while the widths were mostly in the ~70–190 nm range. Figure 1B shows a TEM image of an as-prepared single ZnO nanorod edge with a smooth surface. Figure 1C shows the successful modification of ZnO NRs with irregularly distributed quasi-spherical Ag NPs (~5 to 24 nm). 

In general, the deposition of monodispersed Ag NPs through the chemical surface modification is still a challenging task. On the other hand, the surface plasmon resonance peak of Ag NPs is size-dependent [22,23]; hence, the presence of Ag NPs of various sizes on ZnO NRs can be advantageous in terms of visible light absorbance over a wide range. In fact, UV-Vis measurements (Figure S1, Supplementary Information) confirmed that prepared ZnO-Ag NRs have two distinct absorbance peaks: one in the UV range, which is related to the ZnO absorbance, and another broad peak in the visible range, which is associated with overlapped surface plasmon resonance peaks of various sized Ag NPs [22,23].

**Figure 2:**
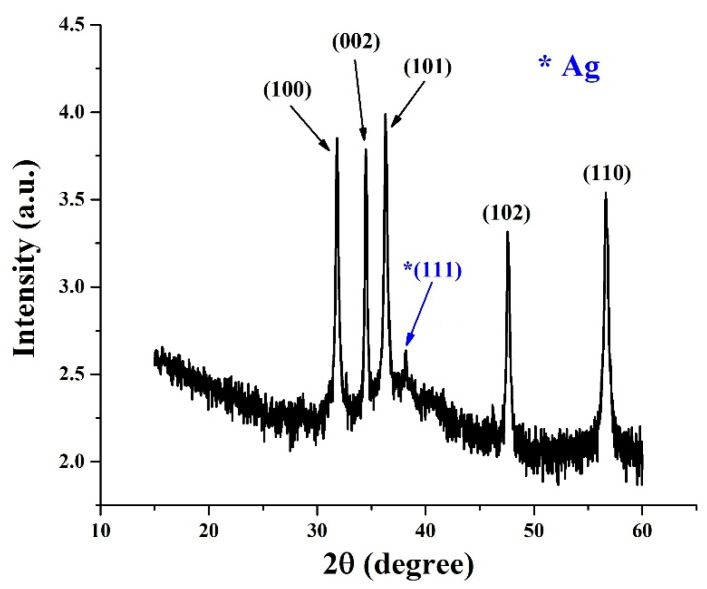
XRD pattern of as-prepared ZnO-Ag NRs.

The successful surface modification of ZnO NRs with Ag NPs was also confirmed by EDX elemental mapping and ICP-OES measurements. The EDX elemental mapping of the selected ZnO-Ag NRs (Figure S2, Supplementary Information) confirmed that Ag NPs are well detectable across the whole surface of ZnO NRs. According to the ICP-OES measurements, the weight of Ag NPs in the ZnO-Ag NRs is ~6.3% wt. XRD was used to further investigate the structural properties of ZnO-Ag NRs. The XRD analysis of as-prepared ZnO-Ag NRs (Figure 2) revealed typical crystalline faces of the wurtzite ZnO structure (JCPDS no. 36-1451), such as (100), (002), (101), (102), and (110) centered at 31.8, 34.5, 36.4, 47.7, and 56.8°, respectively [14,24]. A week peak centered at ~38.4° can be assigned to the (111) facet of Ag (JCPDS no. 04-0783) [14,24]. It should be mentioned that the (200) facet of Ag at ~44.5° is not observable due to low intensity and/or equipment limitations [17,24]. No other peaks are found, indicating that ZnO-Ag NRs were successfully formed.

**Figure 3:**
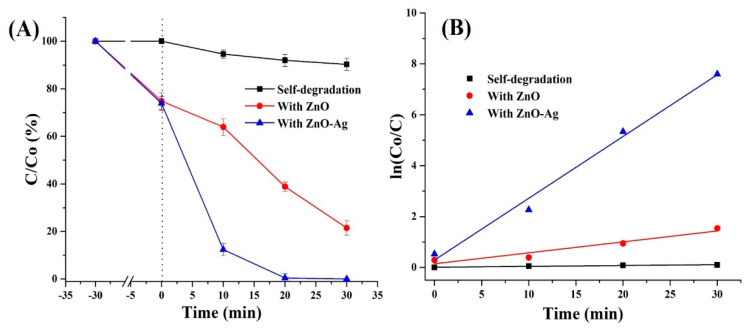
RB dye photodegradation (**A**) and linear fitting (**B**) of ln(*Co/C*) as a function of time.

The photocatalytic activity of bare ZnO and ZnO-Ag NRs was further compared using RB dye as a model pollutant in water. The pH of the RB dye solution was kept at ~7, because ZnO is an amphoteric material that can be degraded at lower or higher pH ranges [24]. Figure 3A depicts the photodegradation kinetics (*C/Co* vs. time) of bare RB dye and in the presence of ZnO and ZnO-Ag NRs. The RB dye concentration remained unchanged after 30 min in the dark, and ~9.7% of RB dye degraded after 30 min of direct solar illumination. In contrast, ZnO and ZnO-Ag NRs adsorbed ~25–26% of the RB dye after 30 min in the dark. However, the photocatalytic activity of both samples was found to be drastically different when exposed to solar light. In particular, after 30 min of illumination, ZnO NRs can degrade ~78.5% of the RB dye, whereas ZnO-Ag NRs can degrade ~99.5% in only 20 min. Corresponding UV-Vis curves are plotted in Figures S3–S5 (Supplementary Information). Figure 3B shows the fitting curves for all three curves. The following equation [25] was used for fitting:

$$\ln\left(\frac{Co}{C}\right)=kt$$

where, *C*—dye concentration at any period of time, *Co*—initial dye concentration, *k*—pseudo-first-rate constant, and *t*—time. The fitting results yield the following mean degradation rates: self-degradation ~0.0034 min^−1^, ZnO ~0.0431 min^−1^, and ZnO-Ag ~0.2432 min^−1^. Hence, it is clear that chemical deposition of Ag NPs can significantly accelerate the photocatalytic activity of ZnO NRs. For example, photodegradation of RB dye in the presence of ZnO-Ag occurs ~71.5 and ~5.6 times faster than self-degradation and ZnO-based photodegradation, respectively.

**Figure 4:**
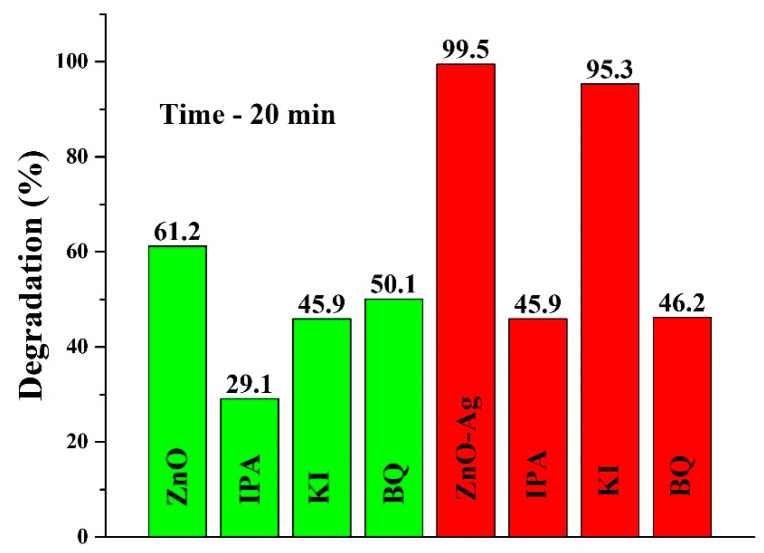
The effects of radical scavengers on RB dye photodegradation.

In the next step, radical scavengers were used to define the active radicals involved in photocatalytic degradation. The observation time was set to 20 min (representing 99.5% RB degradation by ZnO-Ag NRs). Figure 4 depicts the radical scavenging results for ZnO and ZnO-Ag NRs. One can notice the obvious difference, i.e., that without radical scavengers, ZnO and ZnO-Ag NRs can degrade ~61.2 and ~99.5 percent of RB dye, respectively. In the case of ZnO NRs, the degradation percentage rate was significantly decreased (more than two times) with IPA implying that **˙**OH radicals are playing the major role in photodegradation reactions. On the other hand, holes and **˙**O_2_^−^ radicals play a minor role to some extent. When ZnO-Ag photocatalyst is used, the situation changes drastically and holes almost do not participate in dye degradation. However, the degradation percentage is nearly the same with IPA and BQ radical scavengers. Such a situation can occur when **˙**OH and **˙**O_2_^−^ radicals are generated at nearly the same rate. We can speculate that Ag NPs can act as a sink for electrons; hence, holes and electrons can be effectively separated at the interface between ZnO NRs and Ag NPs. The produced electrons are captured by dissolved O_2_ molecules and transformed into superoxide anion (**˙**O_2_^−^) radicals [17]. Meanwhile, holes in the ZnO can react with water molecules and produce **˙**OH radicals. Consequently, the synergetic action of **˙**OH and **˙**O_2_^−^ radicals results in efficient degradation of RB dye. The following reactions represent the overall photodegradation mechanism of RB dye by ZnO-Ag NRs:

h*v* + ZnO-Ag → ZnO(h^+^)-Ag(e^−^)

ZnO(h^+^) + H_2_O → ZnO + **˙**OH + H^+^

Ag(e^−^) + O_2_ → Ag + **˙**O_2_^−^

**˙**OH + **˙**O_2_^−^ + RB → mineralization products (H_2_O + CO_2_)

**Figure 5:**
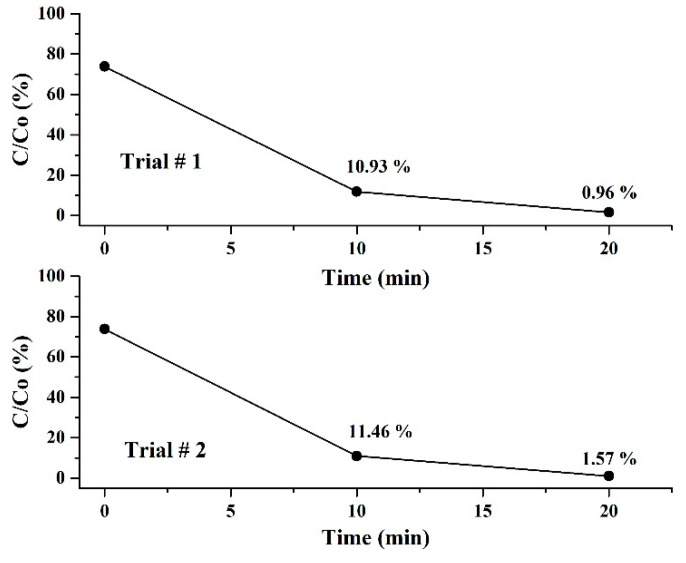
Reusability of ZnO-Ag photocatalysts (second and third run).

Another important factor to consider is photocatalyst reusability in order to decrease treatment costs and prevent secondary pollution. The reusability of the collected ZnO-Ag photocatalyst was tested three times, which were denoted as base trial (first run), trial # 1 (second run), and trial # 2 (third run). Figure 5 depicts how photodegradation decreases with each new trial, i.e., ~99.5% (base trial), ~99.0% (trial # 1), and ~98.4 % (trial # 2). The loss of photodegradation activity can be attributed to a variety of factors, such as overall loss of photocatalysts during the collection process and possible detachment of Ag NPs. Nonetheless, the photocatalytic activity of the ZnO-Ag photocatalyst remains high even after three cycles, indicating its good potential for reusability.

Table 1 compares the efficacy of some recently reported ZnO-based photocatalysts for RB dye degradation. In fact, these results are not directly comparable due to different experimental conditions; however, they may provide a summary of progress in the topic achieved so far. The ZnO-Ag reported in this study appears to be more efficient in terms of dye degradation rate and time required for RB degradation as compared to other ZnO-based photocatalysts. However, there is still room for improvement, for example, one can monitor the fate of APTES in reactions and adjust the Ag shape/concentration, which can further boost the photocatalytic activity [25]. In addition, one can also test the photocatalytic activity of ZnO-Ag NRs with different aspect ratios. Lastly, photocatalytic activity can be studied in the presence of interfering ions. These recommendations can be implemented in future studies.

## 4. Conclusions

In conclusion, we proposed a simple wet chemistry-based method for depositing Ag NPs on the ZnO NRs surface. When compared to bare ZnO NRs, the photocatalytic activity of ZnO-Ag NRs increased significantly (~5.6 times). Experiments with radical scavengers revealed that Ag NPs help with the effective separation of formed charges in ZnO NRs. It was found that the synergetic action of **˙**OH and **˙**O_2_^−^ radicals accelerates the photodegradation of RB dye in solution. We strongly believe that the proposed methodology can be extended to the fabrication of other metal–semiconductor photocatalysts.

## Figures and Tables

**Table 1:** Comparison of ZnO-based catalysts for RB dye photodegradation.

Photocatalyst Type (Mass to Solution Volume Ratio), and Light Source	RB Dye Conc.	Irradiation Time (Degrad. %)	Rate Const., k (min^−1^)	Ref.
Biosynthesized ZnO NPs (1 mg to 5 mL), natural sunlight	1 × 10^−5^ M	200 min (~98%)	0.017	[26]
ZnO/Cu_2_O submicrospheres (1 mg to 1 mL), xenon lamp, 300 W	1 × 10^−5^ M	40 min (~96%)	0.078	[27]
ZnO-Au nanocomposites (1 mg to 0.4 mL), white light ~20 mW/cm^2^)	1 × 10^−5^ M	240 min (~97%)	0.012	[28]
BiOCl/ZnO/CN nanocomposite (1 mg to 5 mL), xenon lamp, 300 W	~4 × 10^−5^ M	20 min (~98.6%)	0.213	[29]
Au-ZnO NRs (1 mg to 0.4 mL), white light ~20 mW/cm^2^)	1 × 10^−5^ M	60 min (~57%)	0.009	[30]
Ag/ZnO@N-carbon composite (1 mg to 2 mL), mercury lamp, 500 W	~1 × 10^−5^ M	25 min (~98.6%)	0.111	[31]
ZnO-Ag NRs (1 mg to 3 mL), solar simulator, 100 W	1 × 10^−5^ M	20 min (~99.5%)	0.243	This work

## Data Availability

Not applicable.

## Supplementary Materials

The following supporting information can be downloaded at: https://www.mdpi.com/article/10.3390/nano12162882/s1, Figure S1: UV-Vis absorbance of ZnO@Ag NRs; Figure S2: EDX elemental mapping of ZnO@Ag NRs; Figure S3: UV-Vis of light-irradiated RB dye (self-degradation); Figure S4: UV-Vis of light-irradiated RB dye (with ZnO); Figure S5: UV-Vis of light-irradiated RB dye (with ZnO-Ag).
