# Supplementary material for: Uncovering the Role of Surface-Attached Ag Nanoparticles in Photodegradation Improvement of Rhodamine B by ZnO-Ag Nanorods
Source: Nanomaterials (Basel). 2022 Aug 22;12(16):2882. doi: 10.3390/nano12162882 (PMC9412419; doi:10.3390/nano12162882)
Supplement: Supplementary file 1 [file nanomaterials-12-02882-s001.zip › nanomaterials-1860947-supplementary.pdf]

Supplementary Information

Uncovering the Role of Surface-Attached Ag Nanoparticles in Photodegradation Improvement of Rhodamine B by ZnO-Ag Nanorods

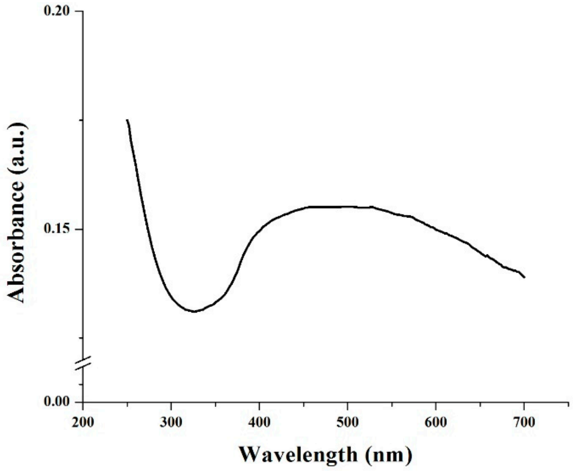

Figure S1. UV-Vis absorbance of ZnO@Ag NRs.

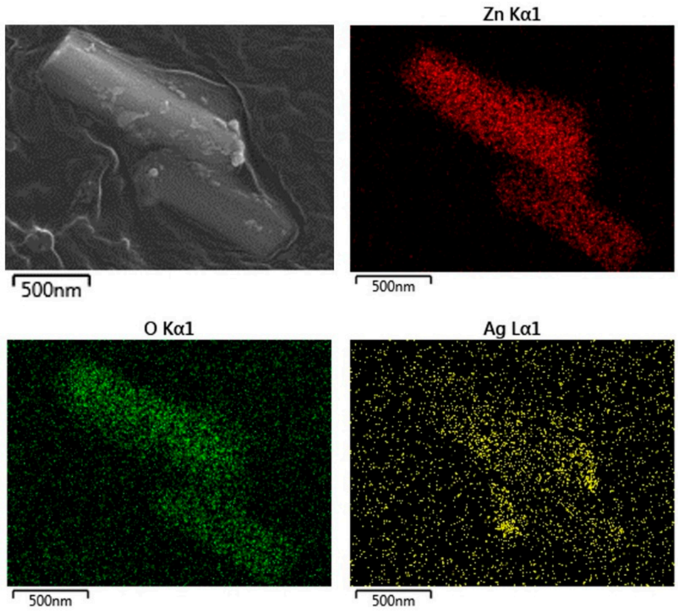

Figure S2. EDX elemental mapping of ZnO@Ag NRs.

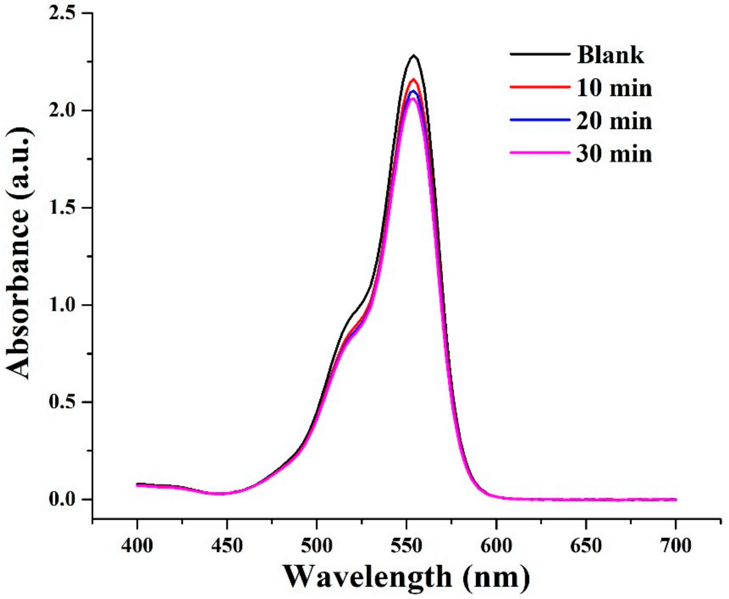

Figure S3. UV-Vis of light-irradiated RB dye (self-degradation).

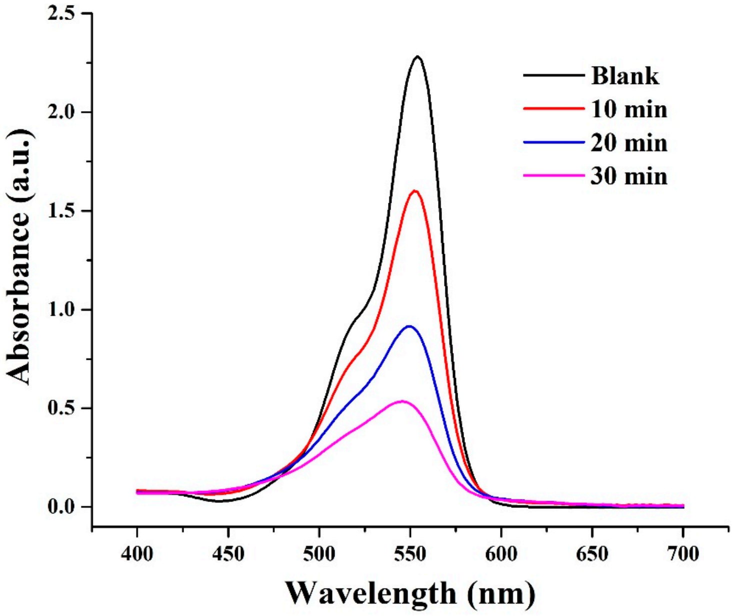

Figure S4. UV-Vis of light-irradiated RB dye (with ZnO).

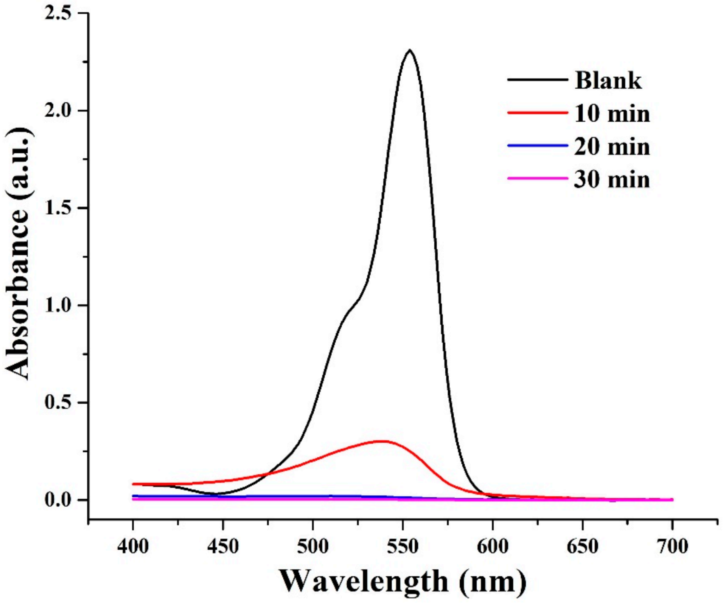

Figure S5. UV-Vis of light-irradiated RB dye (with ZnO-Ag).
